# Inferences about the transmission of lumpy skin disease virus between herds from outbreaks in Albania in 2016

**DOI:** 10.1016/j.prevetmed.2018.12.008

**Published:** 2020-08

**Authors:** Simon Gubbins, Arjan Stegeman, Eyal Klement, Ledi Pite, Alessandro Broglia, José Cortiñas Abrahantes

**Affiliations:** a The Pirbright Institute, Ash Road, Pirbright, Surrey GU24 0NF, UK; b Utrecht University, Department of Farm Animal Health, Utrecht, the Netherlands; c Koret School of Veterinary Medicine, The Hebrew University, Jerusalem, Israel; d Ministry of Agriculture and Rural Development, Sector of Epidemiology and Identification and Registration, Tirana, Albania; e European Food Safety Authority, Via Carlo Magno 1A, 43126 Parma, Italy

**Keywords:** Epidemiology, Cattle, Mathematical modelling, Vaccination, LSDV

## Abstract

Lumpy skin disease has recently emerged as a major threat to cattle populations outside of Africa, where it is endemic. In 2015 the first ever European outbreaks occurred in Greece, which were followed by spread across much of the Balkans in 2016. Here we use a simple mathematical model for the transmission of lumpy skin disease virus (LSDV) between herds to explore factors influencing its spread by fitting it to data on outbreaks in Albania in 2016. We show that most transmission occurs over short distances (<5 km), but with an appreciable probability of transmission at longer distances. We also show that there is evidence for seasonal variation in the force of infection associated with temperature, possibly through its influence on the relative abundance of the stable fly, *Stomoxys calcitrans*. These two results together are consistent with LSDV being transmitted by the bites of blood-feeding insects, though further work is required to incriminate specific species as vectors. Finally, we show that vaccination has a significant impact on spread and estimate the vaccine effectiveness to be 76%.

## 1 Introduction

Lumpy skin disease (LSD) is an economically important disease of cattle and is caused by lumpy skin disease virus (LSDV). Historically, LSD has largely been restricted to Africa (EFSA Panel on Animal Health and Welfare, 2015), but, in recent years, it has spread through the Middle East (into Israel and the Lebanon and reaching Turkey in 2013) and then into south-east Europe, emerging in Greece in 2015. In 2016 LSDV spread to other countries in south-east Europe, including Albania, Bulgaria, the former Yugoslav Republic of Macedonia, Kosovo, Montenegro and Serbia (EFSA, 2017). In the same year LSD also spread along the eastern side of the Black Sea into Armenia, Azerbaijan, Georgia, Russia and Kazakhstan (EFSA, 2017).

Transmission of LSDV is believed to occur principally via the bites of blood-feeding arthropods (Tuppurainen and Oura, 2012; EFSA Panel on Animal Health and Welfare, 2015). This has been demonstrated experimentally for *Aedes aegypti* mosquitos (Chihota et al., 2001) and *Rhipicephalus appendiculatus* male ticks (Tuppurainen et al., 2013), while the stable fly *Stomoxys calcitrans* had been implicated as a vector in Israel by associating seasonality in its abundance with seasonality in LSD outbreaks (Kahana-Sutin et al., 2017). Although the ability of *S. calcitrans* to transmit LSDV has not been shown experimentally, it has been demonstrated to transmit the closely related capripox virus between goats (Mellor et al., 1987). Experimental and field evidence suggests that LSDV is transmitted inefficiently by most other direct or indirect routes (Weiss, 1968; Carn and Kitching, 1995).

Only a few studies have used mathematical modelling to explore the transmission of LSDV within and between herds. Detailed analysis of an outbreak in a cattle herd in Israel in 2006 was used to show the importance of indirect transmission (probably by blood sucking insects) in explaining the observed pattern of spread (Magori-Cohen et al., 2012). Analysis of outbreaks in cattle herds in Ethiopia in 2014–2015 suggested that the basic reproductive ratio (*R*_0_ = 1.1) was only just sufficient to sustain transmission (Molla et al., 2017a), though this is in marked contrast to the estimate of *R*_0_ = 15.7 obtained for the Israeli outbreak (Magori-Cohen et al., 2012). At a regional scale, analysis of reported outbreaks in the Middle East in 2012–2015 indicated that the most important environmental predictors of outbreak location were annual precipitation, mean diurnal temperature range, land cover and livestock density (Alkhamis and VanderWaal, 2016). The authors also estimated the between-herd effective reproduction ratio, which varied from 2.2 to 22.2 depending on region and time of year (Alkhamis and VanderWaal, 2016). Finally, the rate of spread of LSDV in the Balkans in 2015–2016 was estimated to be around 7.3 km/week, but the skewed distribution of the spread rate suggested both local and longer distance spread, possibly related to transmission via vector and animal movements, respectively (Mercier et al., 2018). Following introduction of LSDV, however, most countries in the Balkans implemented vaccination programmes that used the live attenuated Neethling lumpy skin disease vaccine. Vaccination would have influenced the rate of spread, but this was not accounted for in the analysis (Mercier et al., 2018).

In this paper we use a simple model for the transmission of LSDV between herds to address a number of questions about its spread. First, we explore how the force of infection (i.e. the rate at which uninfected herds become infected) depends on the distance between uninfected and infected herds. Second, we assess evidence for seasonality in the force of infection. Third, we estimate the impact of vaccination on the spread of LSDV. We focus our analysis on outbreak data from Albania in 2016 because of the availability of data on cattle demography, on reported outbreaks and on vaccination. In addition, Albania was the most heavily affected country in the Balkans and did not implement stamping out as a control measure, allowing us to avoid any potential biases this could introduce to the analysis.

## 2 Materials and methods

### 2.1 Data

**Fig. 1:**
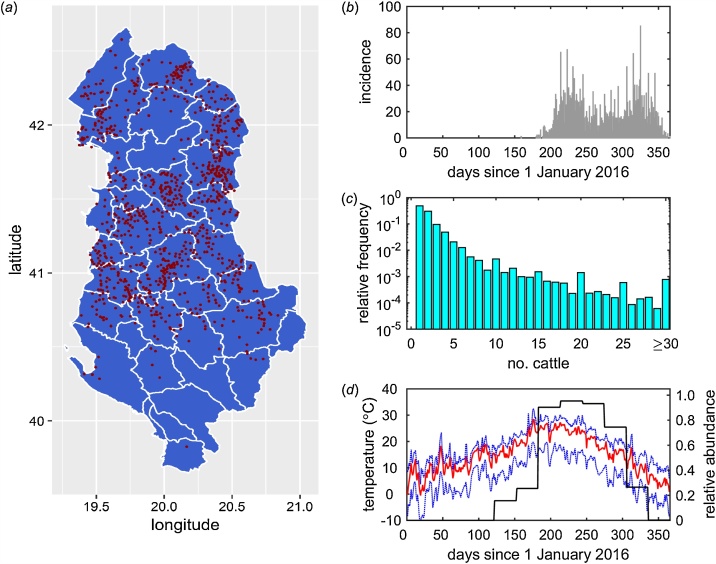
Data used when analysing transmission of lumpy skin disease virus (LSDV) between herds in Albania in 2016. (*a*) Location of reported outbreaks of LSDV. Note that locations are to village level and villages may have had multiple outbreaks. (*b*) Daily incidence of newly reported herds. (*c*) Herd size distribution. (*d*) Median (red line), minimum and maximum (blue dashed lines) daily mean temperatures and mean simulated relative abundance of *Stomoxys calcitrans*, a putative vector of LSDV (black line). (For interpretation of the references to colour in this figure legend, the reader is referred to the web version of this article).

Epidemiological data on reported outbreaks of lumpy skin disease in 2016 were obtained from the Albanian Directorate of Veterinary Services. This provided the location (longitude and latitude; Fig. 1a), date of suspicion (Fig. 1b) and, if known, date of recovery (i.e. when a veterinarian reported there were no more clinically-affected animals in the herd) for each herd which reported lumpy skin disease. In the analyses the date of suspicion was used as a proxy for the date of infection and a herd was considered to be infectious from the date of infection to the date of recovery. If the date of recovery was not known, the herd was assumed to remain infectious until the end of the study period (i.e. 31 December 2016).

The number of cattle (as of February 2017) (Fig. 1c) and the date of vaccination (if vaccinated; all cattle in a herd were assumed to be vaccinated) for all herds in Albania were provided by the Albanian Directorate of Veterinary Services. Location data (latitude and longitude) were not available for individual herds, so the location for the village in which the herd is located was used instead (i.e. herds do not have unique locations).

Temperature data for Albania (Fig. 1d) were obtained from the European Commission Joint Research Centre MARS Meteorological Database (Toreti, 2014), which provides daily meteorological data spatially interpolated on a 25 km by 25 km grid. Specifically, we extracted the daily minimum and daily maximum temperatures for 2016 and computed the midpoint of these for each of the 70 grid cells covering Albania. Herds used the temperatures for the grid cell in which they were located.

### 2.2 Modelling approach

Because we only have data on the location of infected and uninfected herds, we described the transmission of LSDV between herds using a kernel-based approach. In this approach all transmission routes are combined into a single generic mechanism, with the probability of transmission from an infected to an uninfected herd assumed to depend on the distance between them (i.e. the transmission kernel). In addition, we assume the susceptibility of an uninfected herd and the infectiousness of an infected herd are both proportional to the number of cattle in the herd. This type of approach been used when describing the spread of a number of animal diseases, including foot-and-mouth disease (Keeling et al., 2001), classical swine fever (Backer et al., 2009), avian influenza (Boender et al., 2007) and bluetongue (Szmaragd et al., 2009).

**Fig. 2:**
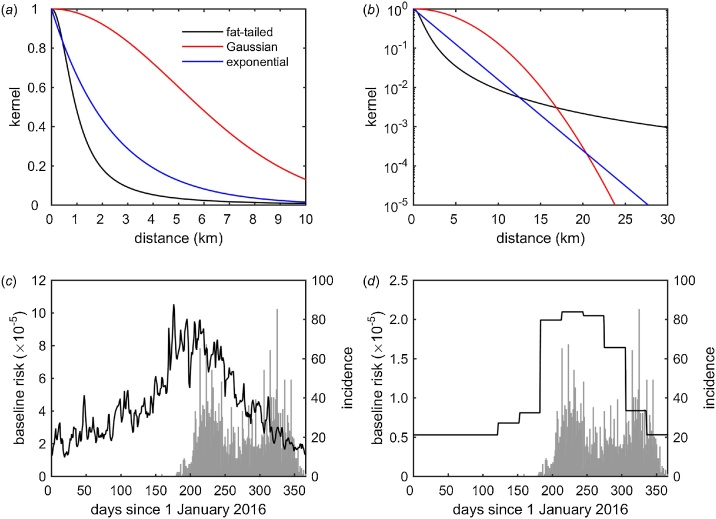
Transmission of lumpy skin disease virus (LSDV) in Albania in 2016. (*a*,*b*) Transmission kernels for LSDV plotted on a (*a*) linear or (*b*) logarithmic scale. (*c*,*d*) Seasonal transmission rate of LSDV when it depends on (*c*) daily mean temperature or (*d*) simulated relative abundance of *Stomoxys calcitrans*, a putative vector of LSDV. The plots show the transmission rate, *h*(*t*) (black line, left-hand axis), and the daily incidence of newly reported herds (grey bars, right axis). The fat-tailed kernel and the transmission rate dependent on relative vector abundance yielded the best fits to the outbreak data. (For interpretation of the references to colour in this figure legend, the reader is referred to the web version of this article).

The force of infection, λ*_i_*(*t*), experienced by herd *i* on day *t* is

(1) $$\lambda_{i}\left(t\right)\;=\;hN_{i}\sum_{j\neq i}K\left(d_{ij}\right)N_{j}I_{j}\left(t\right),$$

where *h* is the transmission rate (i.e. a constant of proportionality, which will encompass a range of epidemiological and environmental factors), *N_i_* and *N_j_* the number of cattle on herds *i* and *j*, respectively, *K*(*d_ij_*) is the distance kernel (see below), *d_ij_* is the great circle distance between herds *i* and *j* and *I_j_*(*t*) is a variable indicating whether herd *j* is uninfected (0) or infected (1) on day *t*. Three different functional forms for the kernel, *K*(*d*), were explored, reflecting different assumptions about how the kernel changes with distance (Fig. 2a,b). These were

(2) $$\begin{array}{l} \text{Fat-tailed}\;\text{kernel}:\;K\;\left(d\right)\;=\;{\left(1\;+\;{\left(\frac{d}{d_{0}}\right)}^{\alpha }\right)}^{-1},\;\\ \text{Gaussian}\;\text{kernel}:\;K\left(d\right)\;=\;\exp\;\left(-{\left(\frac{d}{d_{0}}\right)}^{2}\right),\\ \text{Exponential}\;\text{kernel}:\;K\;\left(d\right)\;=\;\exp\;\left(-\frac{d}{d_{0}}\right), \end{array}$$

where *d*_0_ is the median (fat-tailed) or mean (Gaussian or exponential) distance at which transmission occurs and *α* controls how fat the tails are for the fat-tailed kernel. A key difference between the kernels is that, as its name implies, the fat-tailed kernel allows for a higher probability of transmission at longer distances than either the Gaussian or exponential kernels.

The impact of seasonality was explored in two ways. In the first, the transmission rate, *h*, was assumed to be a function of temperature, so that

(3) $$h\left(t\right)\;=\;\exp\;\left(h_{0}\;+\;h_{1}\;\left(T_{i}\;\left(t\right)\;-\;\bar{T_{i}}\right)\right),$$

where *h*_0_ is the baseline rate, *h*_1_ is the seasonal increase in rate above baseline and *T_i_*(*t*) is the daily mean temperature on day *t* and $\bar{T_{i}}$ is the annual mean temperature at herd *i*. In the second, the transmission rate was assumed to depend on the relative abundance of *S. calcitrans*, one of the putative vectors of LSDV and known to be abundant in the areas where LSDV is circulating in the Balkans. Evidence for *S. calcitrans* being a vector for LSDV comes from its ability to transmit other capripox viruses (Kitching and Mellor, 1986; Mellor et al., 1987) and the strong correlation between abundance of *S. calcitrans* and LSDV outbreaks observed in Israel (Kahana-Sutin et al., 2017). In this case the transmission rate is

(4) $$h\left(t\right)\;=\;\exp\;\left(h_{0}\;+\;h_{1}V_{i}\left(t\right)\right),$$

where *h*_0_ is the baseline rate, *h*_1_ is the seasonal increase in rate above baseline and *V_i_*(*t*) is the relative vector abundance for herd *i* at time *t* (normalised so the maximum is equal to one; Fig. 1d). The relative vector abundance is given by

(5) $$V_{i}\left(t\right)\;=\;cF\;\left(T_{m-1}\right)\;E\;\left(T_{m-1}\right)\;L\;\left(T_{m-1}\right)\;P\;\left(T_{m-1}\right),$$

where *F*, *E*, *L* and *P* are temperature-dependent functions describing fecundity, egg survival, larval survival and pupal survival, respectively, *c* is the normalising constant and *T_m-1_* is the monthly mean temperature for the preceding month. Appropriate functional forms (see Appendix S1 for details) for *F*, *E*, *L* and *P* were obtained from experiments using laboratory colonies of *S. calcitrans* (Lysyk, 1998; Kahana-Sutin et al., 2017).

The effect of vaccination was incorporated in the model by assuming it changes force of infection to reflect: (i) the reduced probability of an unaffected herd becoming infected because of fewer susceptible animals being present; (ii) the reduced infectiousness of an affected herd because of fewer animals becoming infected; and (iii) the reduced infectiousness of an infected, vaccinated animal. To allow for these effects, the force of infection, (1), is changed so that the herd size for an uninfected herd *i* (i.e. *N_i_* in Eq. (1)) is replaced by the number of unprotected animals in the herd at time *t*, that is

(6) $$N_{i}\;\to \;\left(1\;-\;v_{s}\;\left(t\right)\right)\;N_{i},$$

where *v_S_*(*t*) is the vaccine effectiveness for susceptibility at time *t*. In addition, the herd size for infected herd *j* (i.e. *N_j_* in Eq. (1)) is replaced by the number of unprotected cattle at the time the herd was infected multiplied by the reduction in infectiousness for a vaccinated, infected animal, so that

(7) $$N_{j}\;\to \;\left(1\;-\;v_{I}\;\left(t_{I}^{\left(j\right)}\right)\right)\;\left(1-\;v_{s}\;\left(t_{I}^{\left(j\right)}\right)\right)\;N_{j},$$

where *v_I_*(*t*) is the vaccine effectiveness for infectiousness at time *t*, respectively, and $t_{I}^{\left(j\right)}$ is the time at which herd *j* was infected. Vaccine effectiveness was assumed to increase linearly from zero to maximum effectiveness (given by *ε_S_* and *ε_I_* for susceptibility and infectiousness, respectively) at the time full protection is reached (assumed to be 21 days after vaccination as per the manufacturer’s data sheet; MSD Animal Health, 2018). Four possibilities were considered for vaccination: (i) it has no effect (i.e. *v_S_*(*t*) = 0 and *v_I_*(*t*) = 0); (ii) it affects susceptibility only (i.e. *v_I_*(*t*) = 0); (iii) it affects infectiousness only (i.e. *v*_S_(*t*) = 0); or (iv) it affects both susceptibility and infectiousness.

### 2.3 Parameter estimation and model selection

Parameters in the model were estimated using maximum likelihood methods (Pawitan, 2001). The likelihood for the data is given by,

(8) $$\begin{array}{ll} L= & \prod_{i\in U}\exp\left(-\sum_{t=t_{0}}^{t_{end}}\lambda_{i}(t)\right)\times \\ & \prod_{i\in I}\exp\left(-\sum_{t=t_{0}}^{t_{I}^{\left(i\right)}-1}\lambda_{i}(t)\right)\times \left(1-\exp\left(-\lambda_{i}(t_{I}^{\left(i\right)})\right)\right), \end{array}$$

where *U* and *I* are lists of herds which did not or did report cases during the observation period, respectively, *λ_i_*(*t*) is the force of infection defined by Eq. (1), *t*_0_ and *t_end_* are the beginning and end of the observation period, respectively, and *t_I_* is the time at which the herd became infected. The fits of different models (i.e. kernels, assumptions about the effect of vaccination and the effect of seasonality) were compared using the Akaike information criterion (AIC) (Burnham and Anderson, 2002).

## 3 Results

### 3.1 Kernel

A fat-tailed kernel yielded the best fit (ΔAIC>200 for the corresponding models including an exponential or Gaussian kernel), followed by an exponential kernel (ΔAIC>200 for the corresponding model including a Gaussian kernel), with a Gaussian kernel providing the poorest fit. The best fit kernel was independent of the model for seasonality or vaccine effectiveness. Moreover, the estimated kernel parameters did not differ greatly amongst the different models for seasonality or vaccine effectiveness.

The estimated kernel parameters are presented in Table 1 and the kernels are plotted in Fig. 2. All three kernels predict that a majority of transmission occurs at shorter distances (Fig. 2a,b), with the risk of transmission reduced by 95% at a distance of 4.1 km, 7.2 km and 12.1 km for the fat-tailed, exponential and Gaussian kernels, respectively. In addition, the kernels differ in their predictions for the frequency of transmission at longer distances, with the fat-tailed kernel predicting more transmission at distances over 20 km than the exponential or Gaussian ones (Fig. 2b).

**Table 1:** Parameters for transmission between herds and vaccine effectiveness estimated from lumpy skin disease outbreaks in Albania in 2016.

Parameter	Fat-tailed	Gaussian	Exponential
	estimate (95% CI)^a^	estimate (95% CI)	estimate (95% CI)
baseline parameter (*h*_0_)	−12.15 (-12.31, -12.09)	−13.87 (-14.03, -13.72)	−12.65 (-12.82, -12.48)
seasonality parameter (*h*_1_)	1.44 (1.39, 1.49)	1.41 (1.27, 1.57)	1.43 (1.28, 1.60)
distance scale (*d*_0_; km)	0.96 (0.94, 0.99)	7.01 (6.60, 7.46)	2.41 (2.25, 2.58)
kernel parameter (α)	2.02 (2.00, 2.03)	–	–
vaccine effectiveness (%)	76.5 (71.8, 80.6)	76.6 (70.9, 81.5)	77.0 (69.4, 83.2)

a estimate: maximum likelihood estimate; 95% CI: Wald-based 95% confidence interval.

### 3.2 Seasonality

Including seasonality in the force of infection significantly improved model fit (ΔAIC>100 for models without seasonality). Furthermore, a model incorporating seasonality via relative vector abundance provided a better fit than one in which seasonality is incorporated via temperature (ΔAIC>20 for models incorporating seasonality via temperature).

The transmission rate parameters (*h*_0_ and *h*_1_) for the best fit model are presented in Table 1. Comparing the inferred seasonally-varying transmission rate with the incidence of newly-reported cases shows that the initial outbreak coincided with the highest transmission rate (Fig. 2c,d). However, a second peak of newly-reported herds occurred later in the year when the transmission rate was lower (Fig. 2c,d).

### 3.3 Vaccination

Our analysis identified a significant impact of vaccination on spread of LSDV in Albania. In addition, the results indicated that this impact was due to vaccination reducing the susceptibility of an animal, thereby reducing the probability of an unaffected herd becoming infected because of fewer susceptible animals being present (see Eq. (6)) and reducing the infectiousness of an affected herd because of fewer animals becoming infected (see Eq. (7)). More specifically, models in which vaccination affects susceptibility only produced a better fit than models in which it had either no effect or affected infectiousness only (ΔAIC>100). The models in which vaccination affected both susceptibility and infectiousness did not produce a significantly worse fit than those in which it affected susceptibility only (ΔAIC<2), but the simpler model (i.e. susceptibility only) was preferred as it had the smaller number of parameters. The vaccine effectiveness was estimated to be 76% (Table 1).

## 4 Discussion

Using different methods, an earlier analysis of LSDV outbreaks in the Balkans suggested that a majority of transmission occurred at lower rates of spread (around 7.4 km/week), which was attributed to local, vector-borne spread (Mercier et al., 2018). This earlier analysis also identified the occurrence of less frequent, faster transmission at longer ranges (at around 54.6 km/week), which was attributed to movement of infected animals (Mercier et al., 2018). However, this analysis considered only those outbreaks reported up to the end of August 2016, and so did not include all of the outbreaks in Albania (2323 out of 3585 outbreaks occurred after this date). In addition, the analysis implicitly includes the impact of stamping out infected herds on the rate of spread, which was implemented in all affected countries, except Albania. It also did not take into account the effect of vaccination, which was implemented in affected countries only after LSDV was detected and which will have influenced the rate of spread (Mercier et al., 2018). For the exponential or Gaussian kernels fitted to the Albanian outbreak data (Table 1), we can estimate the corresponding rates of spread (i.e. the asymptotic travelling wave speed; Medlock and Kot, 2003), which are 5.8 km/week and 0.9 km/week, respectively. However, the asymptotic travelling wave speed for the fat-tailed kernel is infinite (Kot et al., 1996), meaning that a rate of spread cannot be estimated in this case.

The best-fit kernel for the Albanian outbreak data was fat-tailed with distance scale (*d*_0_) of 0.9 km and kernel parameter (*α*) of 2.0 (Table 1). Analysis of data from an epidemic of LSDV in Israel during 2012–2013 (and using similar methods to the present study) also indicated that a fat-tailed transmission kernel best captured the pattern of spread (and with similar parameter estimates: *α* = 2.01 and *d*_0_ = 1.05 km) (EFSA Panel on Animal Health and Welfare, 2015). The shape of kernel for both Albania and Israel is consistent with LSDV being a vector-borne virus: most transmission occurred over short distances (<5 km), which can be attributed to vector dispersal, but with an appreciable probability of transmission over longer distances, which can be attributed to livestock movements. It is important to note, however, that similar transmission kernels (i.e. with similar distance scales and kernel parameters) have been estimated for directly transmitted viruses, such as foot-and-mouth disease virus (Keeling et al., 2001), classical swine fever virus (Backer et al., 2009) and avian influenza virus (Boender et al., 2007).

A potential source of bias in the analysis of transmission between herds is the location used for each herd, namely the location of the village in which a herd is located rather than the specific location of the herd. Although there are 198,105 herds in Albania, there are only 2938 unique (i.e. village) locations in the data-set, with a median 46 herds with the same location (range: 1–939). However, most herds are small (median herd size is two cattle (range: 1–1000) and the 99th percentile is 15 cattle; Fig. 1b) and, hence, are likely to be clustered within villages (i.e. herds are more likely to be closer to herds in the same village than in another village). Moreover, herds within a village also commonly share grazing. Consequently, the use of village locations for all herds within a village is likely to be a reasonable approximation.

The sensitivity of the estimates for the kernel parameters to using village location was explored by fitting the best-fit model (i.e. one assuming vaccination affects susceptibility only and incorporating seasonality via relative vector abundance) for each of the kernels to outbreak data using synthetic herd locations instead. The synthetic data preserved the clustering of herds within villages, but without assuming all herds have the same location (Appendix S2; Table S1; Fig. S2), nor did the estimates for the transmission rate parameters (*h*_0_ and *h*_1_) or vaccine effectiveness (*ε_S_*) (Table S1).

Previous analyses of LSDV outbreaks have reported seasonality in cases (Alkhamis and VanderWaal, 2016; Kahana-Sutin et al., 2017; Molla et al., 2017b; Mercier et al., 2018), though the timing of peak incidence varies amongst regions. We have found evidence of seasonality in the transmission of LSDV between herds in Albania, which was associated with temperature. More precisely, we have found evidence for a reduction in the force of infection later in the year, which can be ascribed to a reduction in temperature. Because we have used data on cases reported between June and December 2016, the model cannot strictly be used to assess any increase in the force of infection during spring (or indeed any other time). However, the number of reported cases in Albania began increasing in April-May 2017 (EFSA, 2018), which would be consistent with the seasonal force of infection inferred using our model. Following the incursion of LSDV in 2015 the number of reported cases in Greece also showed a seasonal pattern, potentially related to temperature, with an increase in cases starting in April 2016 (EFSA, 2017).

Further analysis of seasonality in transmission in Albania suggested it could be related to the relative abundance of *S. calcitrans*, a putative vector of LSDV. Together with experimental evidence that *S. calcitrans* is able to transmit other capripox viruses (Kitching and Mellor, 1986; Mellor et al., 1987), a similar approach of relating seasonality in LSD outbreaks to abundance of *S. calcitrans* was used to implicate this species as a possible vector of LSDV in Israel (Kahana-Sutin et al., 2017). Clearly, this does not rule out a role for other potential vectors. However, of those arthropod species that are known to be vectors of LSDV, namely *Ae. aegypti* mosquitos (Chihota et al., 2001) and *R. appendiculatus* male ticks (Tuppurainen et al., 2013), *Ae. aegypti* is not known to be present in Albania (ECDC, 2018), while the tick life cycle cannot explain the rapid spread of LSDV in the Balkans.

Although there was evidence of seasonality in transmission, a substantial number of cases were reported in late autumn or winter (Fig. 2c,d). This could reflect possible delays in reporting, but it also suggests that there could be other routes of transmission (i.e. not solely due to arthropod vectors) or additional factors influencing transmission that also need to be taken into account.

A significant impact of vaccination on transmission of LSDV between herds was observed in the outbreak data for Albania. The evidence (based on the change in AIC) was strongest for the vaccine reducing the susceptibility of an animal (and, hence, the susceptibility and infectiousness of a herd). The estimate for vaccine effectiveness was 76.5% (95% CI: 71.8–80.6%) (Table 1). This estimate, obtained from transmission modelling, is higher than that derived for Albania using survival analysis, 62.5% (95% CI: 54.1–69.5) (Klement et al., 2018). Estimates for vaccine effectiveness in other affected countries in the Balkans (using the same vaccine) ranged from 75% (Greece) to 97% (Bulgaria and Serbia) (Klement et al., 2018), which are higher than the estimate for Albania.

In conclusion, the results of this analysis of outbreak data for Albania are consistent with LSDV being vector-borne. This is a consequence of both the range of transmission between herds (typically less than 5 km) and the seasonality of the force of infection being best captured by the seasonality of *S. calcitrans*, a putative vector of LSDV. However, further work is required to incriminate specific species as vectors of LSDV. In particular, detailed trapping on farms is required to give a more complete picture of species composition and seasonality of potential vectors.

## Disclaimer

The present article is published under the sole responsibility of the authors and may not be considered as an EFSA scientific output. The positions and opinions presented in this article are those of the authors alone and do not necessarily represent the views or any official position or scientific works of EFSA. To know about the views or scientific outputs of EFSA, please consult http://www.efsa.europa.eu.

## Conflict of interest

None.
