# Supplementary material for: Inferences about the transmission of lumpy skin disease virus between herds from outbreaks in Albania in 2016
Source: Prev Vet Med. 2020 Aug;181:104602. doi: 10.1016/j.prevetmed.2018.12.008 (PMC7456782; doi:10.1016/j.prevetmed.2018.12.008)
Supplement: Supplementary file 1 [file mmc1.docx]

**Supplementary Material** ***Preventive Veterinary Medicine***

**Inferences about the transmission of lumpy skin disease virus between herds from outbreaks in Albania in 2016**

Simon Gubbins^1^, Arjan Stegeman^2^, Eyal Klement^3^, Ledi Pite^4^, Alessandro Broglia^5^ and José Cortiñas Abrahantes^5^

^1^ *The Pirbright Institute, Ash Road, Pirbright, Surrey GU24 0NF, U.K.*

^2^ *Utrecht University, Department of Farm Animal Health, Utrecht, The Netherlands*

^3^ *Koret School of Veterinary Medicine, The Hebrew University, Jerusalem, Israel*

^4^ *Ministry of Agriculture and Rural Development, Sector of Epidemiology and Identification and Registration, Tirana, Albania*

^5^ *European Food Safety Authority, Via Carlo Magno 1A, 43126 Parma, Italy*

**Appendix S1: Model for relative abundance of *Stomoxys calcitrans***

The relative abundance (*V*) of *Stomoxys calcitrans*, a putative vector of lumpy skin disease virus (LSDV), was described by

$$V_{i}(t)=cF(T_{m-1})E(T_{m-1})L(T_{m-1})P(T_{m-1}),$$

where *F*, *E*, *L* and *P* are temperature-dependent functions describing fecundity, egg survival, larval survival and pupal survival, respectively, *c* is the normalising constant and T*_m_*_-1_ is the monthly mean temperature for the preceding month. Appropriate functional forms for *F*, *E*, *L* and *P* were obtained from experiments using laboratory colonies of *S. calcitrans* (Lysyk, 1998; Kahana-Sutin et al., 2017). Specifically, fecundity (*F*) is given by

$$F(T)=-3523.4+338.6T-6.7T^{2},$$

egg survival (*E*) is given by

$$E(T)=\left\{\begin{matrix} 0 & T<10, \\ 0.31+0.01T+\frac{7.15}{T} & T\geq10, \end{matrix}\right.$$

larval survival (*L*) is given by

$$L(T)=\left\{\begin{matrix} 0 & T<10, \\ 2.39-0.04T-\frac{17.4}{T} & T\geq10, \end{matrix}\right.$$

pupal survival (*P*) is given by

$$P(T)=\left\{\begin{matrix} 0 & T<10, \\ 5.09-0.1T-\frac{40.9}{T} & T\geq10, \end{matrix}\right.$$

with *F* constrained so that *F*>0, and *E*, *L* and *P* constrained so that 0<*E*,*L*,*P*< 1. In addition, *c* was computed so that max(*V*(*t*))=1.

**Appendix S2: Assessing the sensitivity of kernel estimates to location data**

Location data (latitude and longitude) were not available for individual herds, so the location for the village in which the herd is located was used instead (i.e. herds do not have unique locations). To explore the impact of this on parameter estimates and, in particular, those for the kernel, we created a synthetic data-set for Albania in which the herd locations were generated by taking the village location and adding a random perturbation to it for each herd in the village. This preserves the clustering of herds within villages, but without all herds having the same location (Fig. S1).

Specifically, the latitude (*θ_j_*) and longitude (*ϕ_j_*) for herd *j* were perturbed as follows,

where $\theta_{v_{j}}$and are the latitude and longitude of the village (*v_j_*) in which herd *j* is located, respectively, and

$$\begin{matrix} r_{j}\sim U(0,0.02), \\ \psi_{j}\sim U(0,2\pi). \end{matrix}$$

The value 0.02 was chosen so the maximum perturbation distance was around 3 km. The village and synthetic herd locations are shown in Fig. S1.

The model assuming vaccination affects susceptibility only and incorporating seasonality via relative vector abundance was fitted to outbreak data using the synthetic herd locations for the three different kernels (fat-tailed, Gaussian and exponential; see equation (2) in the main paper). The best-fit kernel (as judged by the Akaike information criterion) did not change, with a fat-tailed kernel preferred. Furthermore, the kernel parameters did not change substantially (Table S1; Fig. S2), nor did the estimates for the transmission rate parameters (*h*_0_ and *h*_1_) or vaccine effectiveness (*ε_S_*) (Table S1).

**Table S1.** Comparison of parameter estimates for transmission of lumpy skin disease between herds in Albania using village or synthetic herd locations.

| parameter | fat-tailed | | Gaussian | | exponential | |
| --- | --- | --- | --- | --- | --- | --- |
|  | village | synthetic | village | synthetic | village | synthetic |
| *h*_0_ | -12.15 | -11.81 | -13.87 | -13.94 | -12.65 | -12.75 |
| *h*_1_ | 1.44 | 1.39 | 1.41 | 1.40 | 1.43 | 1.40 |
| *d*_0_ (km) | 0.96 | 0.91 | 7.01 | 7.34 | 2.41 | 2.72 |
| *α* | 2.02 | 2.00 | - | - | - | - |
| *ε_S_* (%) | 76.5 | 76.1 | 76.6 | 76.5 | 77.0 | 76.7 |
| AIC | 32156 | 32448 | 33009 | 33051 | 32486 | 32717 |


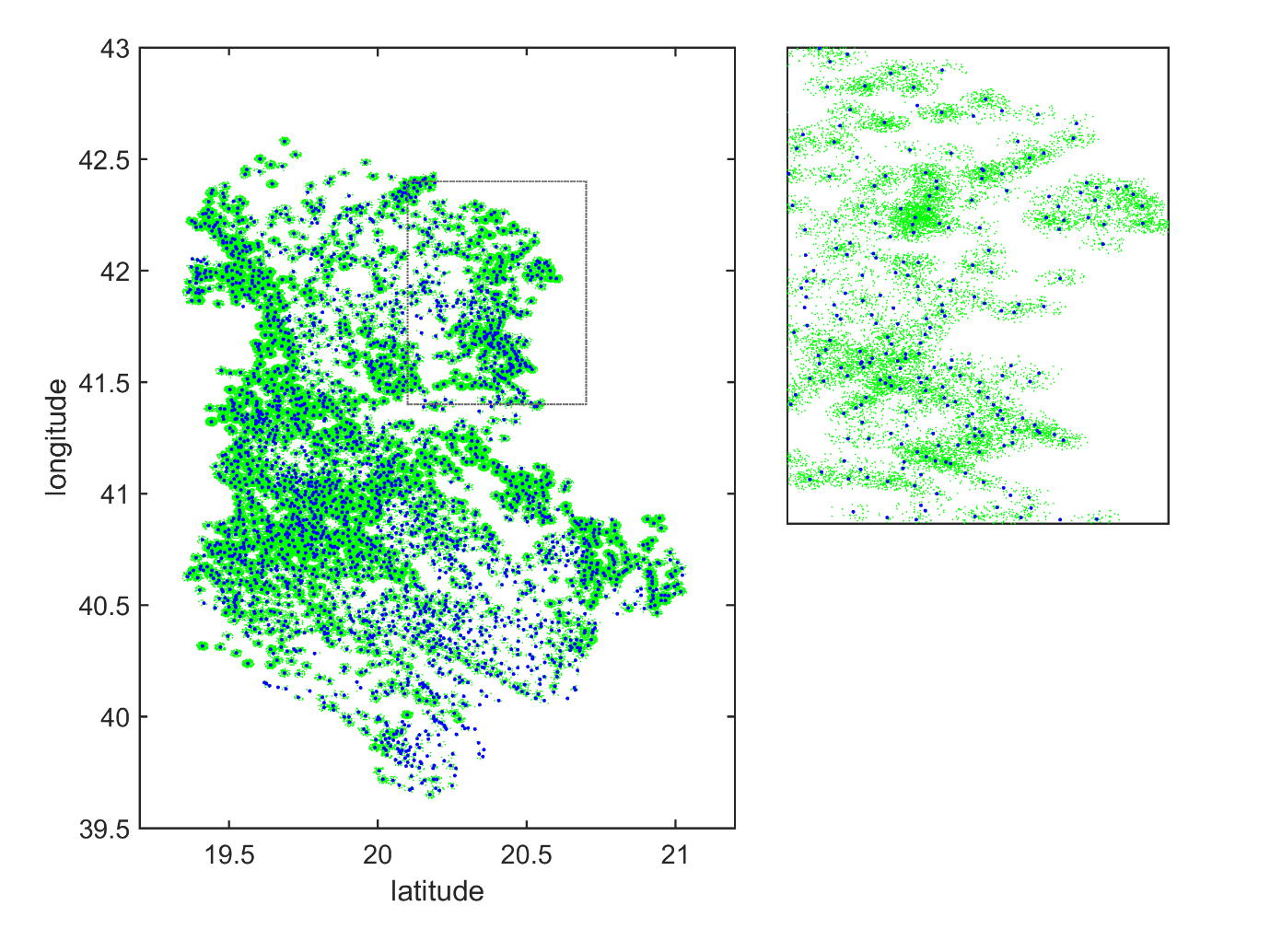


**Fig. S1.** Village and synthetic herd locations for Albania. Blue dots indicate village locations and green dots the synthetic perturbed herd locations. The box in the left-hand panel indicates the area shown in the right-hand panel.


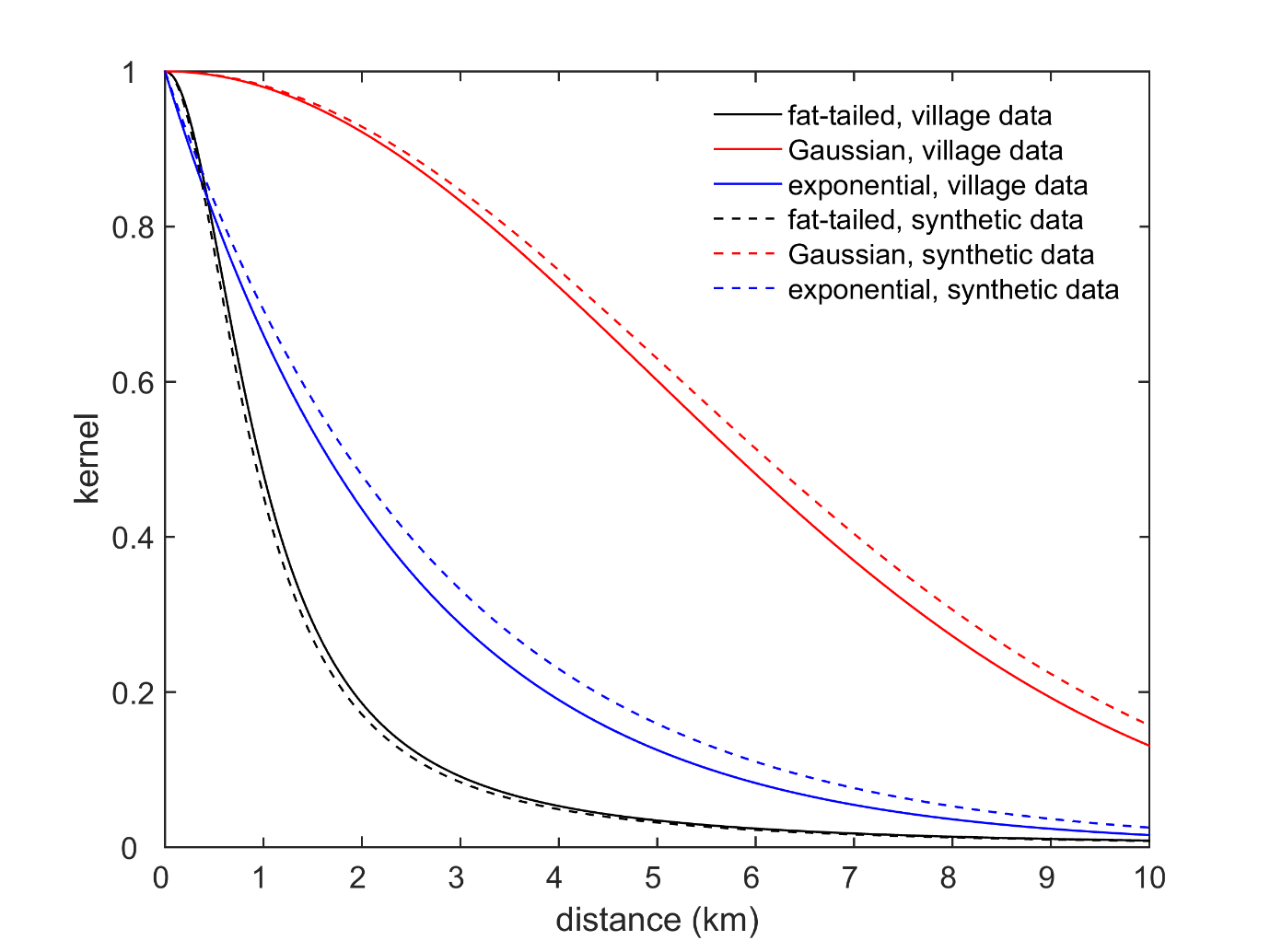


**Fig. S2.** Comparison of transmission kernels for lumpy skin disease virus in Albania estimated from outbreak data using village or synthetic herd locations.
